# No Major Change in vCJD Agent Strain after Secondary Transmission via Blood Transfusion

**DOI:** 10.1371/journal.pone.0002878

**Published:** 2008-08-06

**Authors:** Matthew T. Bishop, Diane L. Ritchie, Robert G. Will, James W. Ironside, Mark W. Head, Val Thomson, Moira Bruce, Jean C. Manson

**Affiliations:** 1 National CJD Surveillance Unit, University of Edinburgh, Western General Hospital, Edinburgh, United Kingdom; 2 Roslin Institute, Neuropathogenesis Division, University of Edinburgh, Edinburgh, United Kingdom; The Scripps Research Institute, United States of America

## Abstract

**Background:**

The identification of transmission of variant Creutzfeldt-Jakob disease (vCJD) by blood transfusion has prompted investigation to establish whether there has been any alteration in the vCJD agent following this route of secondary transmission. Any increase in virulence or host adaptation would require a reassessment of the risk analyses relating to the possibility of a significant secondary outbreak of vCJD. Since there are likely to be carriers of the vCJD agent in the general population, there is a potential for further infection by routes such as blood transfusion or contaminated surgical instruments.

**Methodology:**

We inoculated both wild-type and transgenic mice with material from the first case of transfusion associated vCJD infection.

**Principal Findings:**

The strain transmission properties of blood transfusion associated vCJD infection show remarkable similarities to the strain of vCJD associated with transmission from bovine spongiform encephalopathy (BSE).

**Conclusions:**

Although it has been hypothesized that adaptation of the BSE agent through secondary passage in humans may result in a greater risk of onward transmission due to an increased virulence of the agent for humans, our data presented here in two murine models suggest no significant alterations to transmission efficiency of the agent following human-to-human transmission of vCJD.

## Introduction

Variant Creutzfeldt-Jakob disease (vCJD) is an acquired form of human transmissible spongiform encephalopathy (TSE) caused by infection by the bovine spongiform encephalopathy (BSE) agent that entered the human food chain in the United Kingdom during the 1980s and early 1990s. [1], [2] 164 cases of vCJD have been identified in the United Kingdom and a further 41 cases in other countries worldwide. Annual mortality rates indicate that the vCJD outbreak is now in decline in the UK following a peak in 1999/2000. [3] In 2003 the first case of human-to-human secondary transmission of vCJD via blood transfusion was identified through a collaborative study between the UK National Blood Services, the National CJD Surveillance Unit, and the Office of National Statistics (Transfusion Medicine Epidemiology Review, TMER). [4], [5] Statistical analysis showed that the possibility of this case being due to BSE infection was in the order of 1∶15,000 to 1∶30,000. [4] This patient had received a transfusion of non-leucodepleted red cells that had originated from a donor who 3 years 4 months later developed clinical vCJD. The blood recipient was methionine homozygous at codon 129 of the prion protein (PrP) gene (*PRNP*), the same genotype as all tested vCJD cases. [6]

Two further cases of vCJD linked to blood transfusion, in MM genotype individuals, have subsequently been identified through the TMER study. [7], [8] Following the discovery of these cases policy changes were made in relation to blood donation in the UK and elsewhere. In 2004 the UK Blood Service deferred transfusion recipients from acting as blood donors.

A fourth case, of asymptomatic infection following blood transfusion, was described in 2004 and this individual was heterozygous (MV) at codon 129. [9] This case was the first indication that individuals with *PRNP* genotypes other than MM could be infected by the vCJD agent. All three codon 129 genotypes are now thought to be susceptible to vCJD infection following the identification of two VV genotype appendix tissues positive for vCJD associated PrP (PrP^Sc^) in an anonymous screening study, and the successful transmission of vCJD to ‘humanised’ transgenic mice of each genotype. [10]–[12]

The implications of these findings are that a significant number of the UK population may be carriers of vCJD infectivity, that some of the individuals may be donating blood, and that not only those with an MM genotype may be susceptible to infection from this source. Our research in transgenic models indicates that MV and VV individuals are likely to remain in an infectious preclinical state for a significant period of time with incubation periods potentially longer than average lifespan. [12] The identification of four instances of secondary transmission of vCJD infection from a group of 66 individuals known to have received blood products from vCJD donors, including only 28 who survived at least five years post transfusion indicates that blood transfusion is a significant risk factor for vCJD. This is likely to be due to either the route of transmission being more efficient of the agent being more infectious on human-to-human transmission or a combination of both.

TSE transmission by the blood transfusion route has been investigated in a sheep model. [13], [14] These studies used intravenous (i.v.) transfusion of whole blood and blood fractions from clinical and preclinical sheep infected with BSE or scrapie. Preliminary data showed that the i.v. route gave relatively short and consistent incubation periods suggesting an efficient transmission route, with success rates of 60% for sheep infected with BSE and 40–45% for natural scrapie. [14], [15]

Strain characterisation using a standard panel of inbred lines of wild-type mice originally demonstrated that BSE and vCJD agents had similar biological properties following transmission. [2], [16] Similar work in other murine models has also been undertaken to study other human TSEs (genetic and iatrogenic CJD [17], and sporadic CJD [2]), and has been used to examine emerging TSEs (atypical BSE [18] and chronic wasting disease in deer and elk [19]). [20] The development of transgenic mice expressing human PrP has lead to further dissection of the nature of human TSE strains, including transmission of vCJD to gene targeted human transgenic mice. [12], [17], [21], [22] Extensive data from studies in both wild-type and transgenic models at the Neuropathogenesis Division provide an essential background which will allow us to identify any change in the transmission characteristics of vCJD following secondary transmission. [2], [12], [23]

To investigate the nature of the transmissible agent following secondary transmission from human-to-human following blood transfusion we have examined the biological properties of brain material from the first case of transfusion-associated vCJD inoculated into panels of both wild-type, and transgenic mice expressing human PrP.

## Results

**Figure 1:**
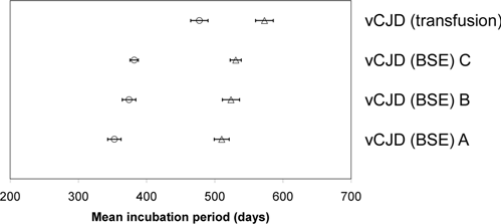
Comparison of incubation periods in wild-type mice. Incubation period plot comparison of vCJD (transfusion) case versus transmissions in wild-type mice of vCJD (BSE) from three sources. (Data shows mean incubation period±standard error of the mean. Open circles RIII line and open triangles VM line.)

Clinical signs of a TSE in the transgenic mice were rare and occurred after long incubation periods (IP) as found in our previous study. [12] Inoculation of the vCJD (transfusion) case produced one clinically positive HuMM mouse (at 659 days post inoculation), two positive HuMV mice (at 596 and 638 dpi) and no positive HuVV mice. Transmission of the vCJD (transfusion) case to the RIII and VM lines showed extended incubation periods compared to the three vCJD (BSE) cases. However, the hierarchy of incubation periods in the two wild-type lines was identical. (Figure 1 and Table 1) These data also show close similarities to previously published vCJD (BSE) transmission to wild-type mice despite different methodologies. These earlier studies used cerebellar material for the inoculum which was injected by simultaneous intracerebral and intraperitoneal routes. [2], [23], [24]

**Table 1:** Clinical and pathological assessment of wild-type mice.

Inoculum	Mouse Line	Mice Inoculated^a^	Positive for Clinical TSE Signs	Positive for TSE Vacuolation	Incubation Period (days±SEM)
vCJD(BSE) A	RIII	20	17	17	352.76±9.78
vCJD(BSE) B	RIII	20	18	17	374.35±9.98
vCJD(BSE) C	RIII	21	17	16	381.88±6.07
vCJD (transfusion)	RIII	23	18	18	477.33±12.68
vCJD(BSE) A	VM	22	15	22	510.20±10.97
vCJD(BSE) B	VM	22	20	21	523.75±12.57
vCJD(BSE) C	VM	21	13	18	530.69±8.16
vCJD (transfusion)	VM	22	15	18	572.90±12.96

Wild-type mouse lines RIII and VM, inoculated with vCJD(BSE) and vCJD(transfusion) were assessed clinically and pathologically for signs of TSE and mean incubation periods calculated.

a The group of 24 was reduced due to unavailability of some brain material for analysis.

**Figure 2:**
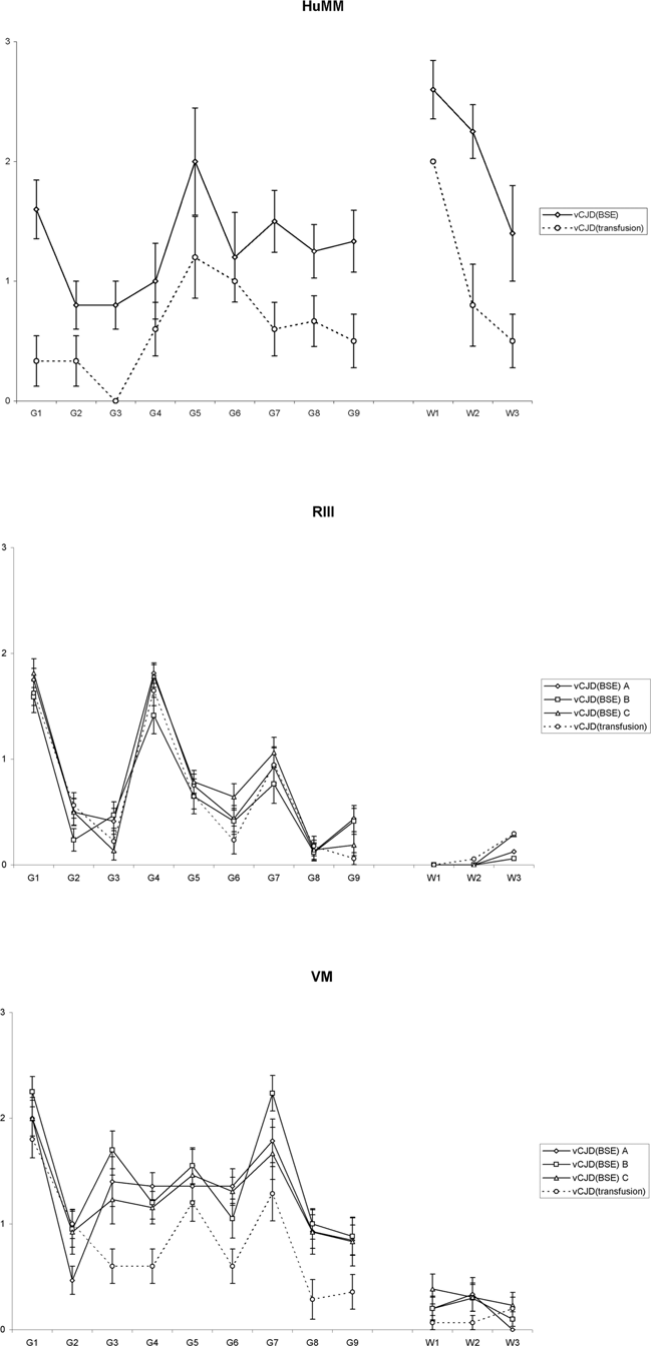
Vacuolation scoring in the mouse brain. Lesion profile comparison of vCJD (transfusion) case versus vCJD (BSE) transmissions to identify similarities in vacuolar pathology levels and regional distribution in mouse brains. (mean score ±SEM; dashed line - vCJD (transfusion) case; solid lines – 3x vCJD (BSE) cases for wild-type mice (diamonds – vCJD(BSE) A; squares – vCJD(BSE) B; triangles – vCJD(BSE) C) and published vCJD (BSE) for HuMM transgenic; G1–G9 grey matter scoring regions; W1–W3 white matter scoring regions)

The frequency of transgenic mice positive for TSE associated vacuolation was similar between the vCJD (transfusion) case and the published vCJD (BSE) case [12], with positive results in 8/15 HuMM, 0/17 HuMV, and 0/17 HuVV mice and 6/16 HuMM, 1/15 HuMV, and 1/15 HuVV mice respectively. Regional distribution of TSE vacuolation in the brain was assessed through lesion profiling. All wild-type and the HuMM transgenic lines had sufficient positive mice to generate a profile (n≥6 mice). The overall pattern of the lesion profiles was the same in the vCJD (transfusion) and vCJD (BSE) cases for all lines of mice, however, for the former case the VM and HuMM mice scores were lower. (Figure 2)

**Figure 3:**
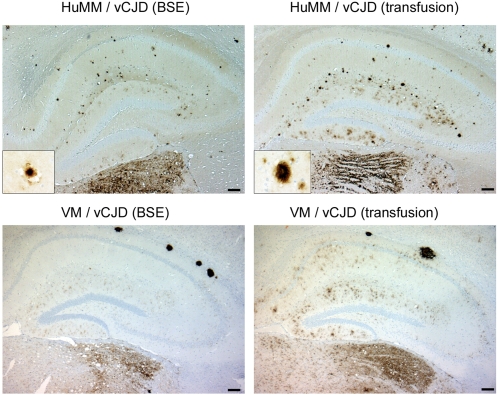
Detection of abnormal PrP in the mouse brain. Immunocytochemical detection of abnormal PrP deposition in hippocampus and thalamus (lateral posterior nucleus) of HuMM transgenic (with additional 40× magnification of florid plaque structure, see box lower left) and VM wild-type mice following inoculation with vCJD (BSE) and vCJD (transfusion) material. (Scale bar 200 µm, anti-PrP antibody 6H4)

Immunocytochemical (ICC) detection of disease associated abnormal PrP in paraffin sections was also used as a method of assessing whether mice were transmission positive. There were 13/14 HuMM, 8/17 HuMV, 1/17 HuVV positive mice in the vCJD (transfusion) case, which was similar to the frequency of positives in the published vCJD (BSE) case: 11/15 HuMM, 11/13 HuMV, 1/15 HuVV mice. ICC data can be used to show variation in targeting of abnormal PrP deposition in the brain and variation in the nature of deposits. The ICC pattern in transgenic mice inoculated with the vCJD (transfusion) case matched that reported for vCJD (BSE) [12]. The thalamus was specifically targeted with deposition of abnormal PrP, and for the HuMM mice the hippocampus contained many intensely stained plaques including vCJD transmission associated florid plaques. ICC pattern in wild-type mice also showed similarities between the data sets with abnormal PrP deposition targeted to the thalamus and hippocampus, and large aggregates in the white matter of the corpus callosum. (Figure 3)

**Figure 4:**
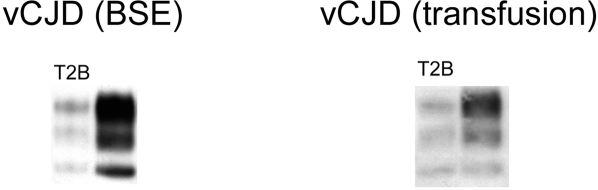
PrP^Sc^ typing by Western blot. Brain homogenates from HuMM mice inoculated with both vCJD (BSE) and vCJD (transfusion) show similar mobility and glycosylation profile (type 2B) as material from vCJD patients. (T2B: control vCJD material; antibody: 6H4)

Biochemical analysis of disease-associated PrP by Western blot can discriminate between human cases of vCJD and sporadic CJD. [25] In the vCJD (transfusion) case the HuMM mice had a type 2B gel mobility and glycoform ratio identical to that found in vCJD (BSE) transmission to HuMM mice, and in vCJD itself. (Figure 4) Brain tissue from both vCJD (transfusion) [4] and published vCJD (BSE) [26] patients showed the type 2B pattern. The levels of PrP^Sc^ seen in the HuMV and HuVV were too low to allow typing by this standard Western blot method.

## Discussion

Secondary passage of vCJD infection via blood transfusion in an MM codon 129 genotype individual results in a clinical disease phenotype and pathological characteristics that are similar to vCJD derived from BSE. [4] In this paper we confirm that the agent strain properties of primary and secondary vCJD cases are similar in transmission studies in transgenic and wild-type mice. Strain characteristics can be assessed by the frequency of clinical signs in recipient animals, the incubation period, neuropathological features, and PrP typing. All these parameters were similar in the transmission studies of primary and secondary vCJD in transgenic mice, indicating that the strain properties of the vCJD agent have not changed significantly following secondary passage in humans.

There were some differences in the results of the transmission studies which deserve further comment. The incubation period in wild-type mice was relatively extended in the vCJD(transfusion) case. However, the hierarchy of incubation periods in different inbred mouse strains was unchanged and the most plausible explanation for these findings is that, rather than implicating a change in agent characteristics, the titre of infectivity was less in the brain sample from the vCJD(transfusion) case. The distribution and degree of vacuolation was identical in the RIII mice. (Figure 2) While the distribution was identical in the VM and HuMM mice the degree of vacuolation intensity was lower for the vCJD(transfusion) case. This variability could be due to the much longer incubation times observed in these lines of mice or due to minor changes of the strain properties.

Preliminary investigation of the individuals diagnosed with vCJD following blood transfusion does not indicate a change in the neuropathological characteristics of vCJD following secondary transmission, although further studies are required to confirm this observation.

The level of infectivity in peripheral tissues in secondary cases of vCJD is unknown, although spleen and a lymph node were PrP positive in the sub-clinical case linked to blood transfusion. Evidence from BSE inoculation of primates indicates similar peripheral distribution of disease associated PrP following either oral or intravenous infection. [27] Further studies are required to assess the anatomical distribution, strain properties and level of infectivity in peripheral tissues in secondary vCJD infection. This may be important for accurate assessment of the public health risks associated with the potential for iatrogenic transmission of vCJD, which are not solely defined by the agent characteristics in brain.

Blood transfusion appears to be a relatively efficient means of secondary transmission of vCJD. To date, there have been four such transmissions in a cohort of 28 individuals who survived at least five years following transfusion of blood derived from individuals incubating vCJD. Despite extensive exposure of the UK population to the BSE agent in the food chain, there have been a relatively limited number of primary cases of vCJD (164 in the UK) and the outbreak has been in decline since 1999/2000. An important question is why there should be a disparity in the apparent efficiency of infection between primary and secondary vCJD. Transmission is generally more efficient within species than between species which may explain this observation. [28], [29] Inoculation of wild-type mice with material from primary and secondary BSE passage in macaques showed that the BSE agent retained a characteristic lesion profile even though the second passage incubation period in the macaques was reduced by 50%. [30] This suggests that efficiency of transmission may increase without obvious changes to the agent strain.

Another factor is that the intravenous route of infection is very much more efficient than the oral route, as shown in experimental models. [27], [31], [32] Results from this study suggest the major factor here is likely to be the route of infection rather than any changes in the strain of agent. Future studies, including those using experimental oral exposure to infectivity in transgenic mice, will further address this issue.

All the primary and secondary clinical cases of vCJD have occurred in individuals with a MM genotype. The sub- or pre-clinical transfusion related infection was in a codon 129 heterozygote and genotyping of positive appendix samples identified in a screening study confirmed valine homozygosity in 2 of 3 samples tested. [10] This indicates that individuals with all codon 129 genotypes may be infected with the vCJD agent and the effect of the MV or VV background on the characteristics of the vCJD agent have not been addressed by the data in this paper.

In conclusion, transmission studies indicate that the strain characteristics of vCJD have not been significantly altered by secondary transmission through blood transfusion. This suggests that the risk of onward transmission of vCJD through other routes, for example contaminated surgical instruments, have not been increased by adaptation of the infectious agent to humans following secondary passage. However the characteristics of the infectious agent in different genetic backgrounds has not yet been defined and the prevalence of vCJD infection in the general population remains uncertain. There is need to continue to implement appropriate policies to protect against the risk of secondary transmission of vCJD until many of the remaining uncertainties are resolved.

## Materials and Methods

The transgenic mice (HuMM, HuMV, HuVV) used in these experiments have been described previously. [12] These mice express human PrP under the regulation of the murine promoter sequences, and survive for the same lifespan as non-transgenic mice of the same genetic background (129Ola) with no adverse effects and no features of spontaneous TSE disease. Wild-type mice (lines VM and RIII) are inbred lines used routinely for strain typing of TSEs. RIII is a *Prnp*-a genotype line and VM is a *Prnp*-b genotype line. [33] Use of mice for this work was reviewed and approved by the Neuropathogenesis Division Ethics Committee for Animal Experimentation.

Mice were inoculated as described previously. Groups of 24 wild-type mice received a 0.02 ml dose at 10^−1^ dilution by the intracerebral route, for vCJD (transfusion) and vCJD (BSE). Groups of 18 transgenic mice were injected with inoculum at a higher dilution of 10^−2^ as in previous experiments more concentrated inocula had been found to be toxic to the mice. Inoculum was prepared as a homogenate in sterile saline from frozen frontal cortex (with full consent from the patient's relatives, and approved by the Lothian NHS Board Research Ethics Committee (Reference: 2000/4/157)) to allow accurate comparison with previous data. Cases used for transmission were: the first blood transfusion associated case, designated here as vCJD (transfusion), and three historical vCJD cases designated here as vCJD (BSE) A, B, and C. The historical vCJD cases were not inoculated into the transgenic mice. Data from vCJD (transfusion) inoculation of the transgenic mice was compared with that already published for vCJD (BSE). [12] Data from vCJD (transfusion) inoculation of the wild-type mice was compared with data from the three historical vCJD cases.

Mice were housed in independently ventilated cages in a Category 3 facility, monitored daily and scored for signs of TSE disease weekly from 100 days post inoculation. Mice were culled, when clinical TSE was evident or for animal welfare reasons, by cervical dislocation and the brain bisected sagittally; one half frozen for biochemical analysis of disease-associated prion protein and the other half fixed in formalin for histology.

Vacuolation scoring was performed according to published protocols and lesion profiles generated. [34], [35] Immunocytochemical detection of abnormal PrP deposition was performed as published and Western blotting of disease-associated PrP from the frozen half-brain carried out according to Head *et al*. [12], [25]

## References

[1] Will RG, Ironside JW, Zeidler M, Cousens SN, Estibeiro K (1996). A new variant of Creutzfeldt-Jakob disease in the UK.. Lancet.

[2] Bruce ME, Will RG, Ironside JW, McConnell I, Drummond D (1997). Transmissions to mice indicate that ‘new variant’ CJD is caused by the BSE agent.. Nature.

[3] Andrews NJ, Farrington CP, Ward HJ, Cousens SN, Smith PG (2003). Deaths from variant Creutzfeldt-Jakob disease in the UK.. Lancet.

[4] Llewelyn CA, Hewitt PE, Knight RS, Amar K, Cousens S (2004). Possible transmission of variant Creutzfeldt-Jakob disease by blood transfusion.. Lancet.

[5] Hewitt PE, Llewelyn CA, Mackenzie J, Will RG (2006). Creutzfeldt-Jakob disease and blood transfusion: results of the UK Transfusion Medicine Epidemiological Review study.. Vox Sang.

[6] Zeidler M, Stewart G, Cousens SN, Estibeiro K, Will RG (1997). Codon 129 genotype and new variant CJD.. Lancet.

[7] HPA (2006). New case of blood transfusion-associated variant-CJD.. CDR Weekly.

[8] Wroe SJ, Pal S, Siddique D, Hyare H, Macfarlane R (2006). Clinical presentation and pre-mortem diagnosis of variant Creutzfeldt-Jakob disease associated with blood transfusion: a case report.. Lancet.

[9] Peden AH, Head MW, Ritchie DL, Bell JE, Ironside JW (2004). Preclinical vCJD after blood transfusion in a PRNP codon 129 heterozygous patient.. Lancet.

[10] Ironside JW, Bishop MT, Connolly K, Hegazy D, Lowrie S (2006). Variant Creutzfeldt-Jakob disease: prion protein genotype analysis of positive appendix tissue samples from a retrospective prevalence study.. BMJ.

[11] Hilton DA, Ghani AC, Conyers L, Edwards P, McCardle L (2004). Prevalence of lymphoreticular prion protein accumulation in UK tissue samples.. J Pathol.

[12] Bishop M, Hart P, Aitchison L, Baybutt H, Plinston C (2006). Predicting susceptibility and incubation time of human-to-human transmission of vCJD.. The Lancet Neurology.

[13] Houston F, Foster JD, Chong A, Hunter N, Bostock CJ (2000). Transmission of BSE by blood transfusion in sheep.. Lancet.

[14] Hunter N, Foster J, Chong A, McCutcheon S, Parnham D (2002). Transmission of prion diseases by blood transfusion.. J Gen Virol.

[15] McCutcheon S, Hunter N, Foster JD, Macgregor I, Hornsey V (2007). Transmission of BSE infection in sheep via blood transfusion.

[16] Hill AF, Desbruslais M, Joiner S, Sidle KCL, Gowland I (1997). The same prion strain causes vCJD and BSE.. Nature.

[17] Taguchi Y, Mohri S, Ironside JW, Muramoto T, Kitamoto T (2003). Humanized knock-in mice expressing chimeric prion protein showed varied susceptibility to different human prions.. Am J Pathol.

[18] Capobianco R, Casalone C, Suardi S, Mangieri M, Miccolo C (2007). Conversion of the BASE Prion Strain into the BSE Strain: The Origin of BSE?. PLoS Pathogens.

[19] Kong Q, Huang S, Zou W, Vanegas D, Wang M (2005). Chronic wasting disease of elk: transmissibility to humans examined by transgenic mouse models.. J Neurosci.

[20] Manson JC, Cancellotti E, Hart P, Bishop MT, Barron RM (2006). The transmissible spongiform encephalopathies: emerging and declining epidemics.. Biochem Soc Trans.

[21] Korth C, Kaneko K, Groth D, Heye N, Telling G (2003). Abbreviated incubation times for human prions in mice expressing a chimeric mouse-human prion protein transgene.. Proc Natl Acad Sci U S A.

[22] Asante EA, Linehan JM, Gowland I, Joiner S, Fox K (2006). Dissociation of pathological and molecular phenotype of variant Creutzfeldt-Jakob disease in transgenic human prion protein 129 heterozygous mice.. Proc Natl Acad Sci U S A.

[23] Bruce ME, McConnell I, Will RG, Ironside JW (2001). Detection of variant Creutzfeldt-Jakob disease infectivity in extraneural tissues.. Lancet.

[24] Bruce M, Will R, Ironside J, Fraser H, Hornlimann B, Riesner D, Kretzschmar H (2006). Evidence for a link between variant Creutzfeldt-Jakob disease and bovine spongiform encephalopathy.. Prions in Humans and Animals.

[25] Head MW, Bunn TJ, Bishop MT, McLoughlin V, Lowrie S (2004). Prion protein heterogeneity in sporadic but not variant Creutzfeldt-Jakob disease: U.K. cases 1991–2002.. Ann Neurol.

[26] Cooper JK, Ladhani K, Minor PD (2007). Reference materials for the evaluation of pre-mortem variant Creutzfeldt-Jakob disease diagnostic assays.. Vox Sang.

[27] Herzog C, Sales N, Etchegaray N, Charbonnier A, Freire S (2004). Tissue distribution of bovine spongiform encephalopathy agent in primates after intravenous or oral infection.. Lancet.

[28] Baron T (2002). Mouse models of prion disease transmission.. Trends Mol Med.

[29] Bruce M, Chree A, McConnell I, Foster J, Pearson G (1994). Transmission of bovine spongiform encephalopathy and scrapie to mice - strain variation and the species barrier.. Philosophical Transactions of the Royal Society of London Series B-Biological Sciences.

[30] Lasmezas CI, Fournier JG, Nouvel V, Boe H, Marce D (2001). Adaptation of the bovine spongiform encephalopathy agent to primates and comparison with Creutzfeldt– Jakob disease: implications for human health.. Proc Natl Acad Sci U S A.

[31] Cervenakova L, Yakovleva O, McKenzie C, Kolchinsky S, McShane L (2003). Similar levels of infectivity in the blood of mice infected with human-derived vCJD and GSS strains of transmissible spongiform encephalopathy.. Transfusion.

[32] Bartz JC, Kincaid AE, Bessen RA (2003). Rapid prion neuroinvasion following tongue infection.. J Virol.

[33] Barron RM, Baybutt H, Tuzi NL, McCormack J, King D (2005). Polymorphisms at codons 108 and 189 in murine PrP play distinct roles in the control of scrapie incubation time.. J Gen Virol.

[34] Fraser H, Dickinson AG (1968). The sequential development of the brain lesion of scrapie in three strains of mice.. Journal of Comparative Pathology.

[35] Bruce ME, McConnell I, Fraser H, Dickinson AG (1991). The disease characteristics of different strains of scrapie in Sinc congenic mouse lines: implications for the nature of the agent and host control of pathogenesis.. J Gen Virol.

